# Singapore Grouper Iridovirus (SGIV) Inhibited Autophagy for Efficient Viral Replication

**DOI:** 10.3389/fmicb.2020.01446

**Published:** 2020-06-26

**Authors:** Chen Li, Liqun Wang, Jiaxin Liu, Yepin Yu, Youhua Huang, Xiaohong Huang, Jingguang Wei, Qiwei Qin

**Affiliations:** ^1^Joint Laboratory of Guangdong Province and Hong Kong Region on Marine Bioresource Conservation and Exploitation, College of Marine Sciences, South China Agricultural University, Guangzhou, China; ^2^Guangdong Laboratory for Lingnan Modern Agriculture, Guangzhou, China; ^3^Laboratory for Marine Biology and Biotechnology, Qingdao National Laboratory for Marine Science and Technology, Qingdao, China

**Keywords:** SGIV, grouper, autophagy, LC3, Atg5, p53

## Abstract

Autophagy is a conserved catabolic process that occurs at basal levels to maintain cellular homeostasis. Most virus infections can alter the autophagy level, which functions as either a pro-viral or antiviral pathway, depending on the virus and host cells. Singapore grouper iridovirus (SGIV) is a novel fish DNA virus that has caused great economic losses for the marine aquaculture industry. In this study, we found that SGIV inhibited autophagy in grouper spleen (GS) cells which was evidenced by the changes of LC3-II, Beclin1 and p-mTOR levels. Further study showed that SGIV developed at least two strategies to inhibit autophagy: (1) increasing the cytoplasmic p53 level; and (2) encoding viral proteins (VP48, VP122, VP132) that competitively bind autophagy related gene 5 and mediately affect LC3 conversion. Moreover, activation of autophagy by rapamycin or overexpressing LC3 decreased SGIV replication. These results provide an antiviral strategy from the perspective of autophagy.

## Introduction

Singapore grouper iridovirus (SGIV) is a novel marine fish virus isolated from diseased groupers (Qin et al., 2001). The clinical symptoms of SGIV-challenged fishes are hemorrhage and enlargement of the spleen. This lethal pathogen has caused considerable economic damage in groupers, with more than 90% mortality (Qin et al., 2003).

To date, the morphogenesis, biochemical pathology, genome, transcriptome, proteomics, and entry mechanisms of SGIV have been systematically studied (Qin et al., 2003; Song et al., 2004; Wang et al., 2014). SGIV is an icosahedral virus with diameter of 154–176 nm, and it belongs to the genus *Ranavirus* and family Iridoviridae (Qin et al., 2001, 2003). The entire SGIV genome is a double-stranded DNA that consists of 140,131 base pairs and codes 162 open reading frames (ORFs) (Song et al., 2004). Among them, the function of some important viral proteins has been explored. For example, ORF136 encodes a lipopolysaccharide-induced tumor necrosis factor (TNF)-α factor (LITAF) homolog, and ORF051 encodes TNF receptor homologs and functions as a critical virulence factor that is involved in apoptosis and virus-mediated immune evasion (Huang et al., 2008; Yu et al., 2017). Study of the unknown viral genes will provide clues to its pathogenic mechanism as well as information about host–pathogen interactions, especially the precise strategy by which viruses escape the host immune response.

Autophagy is a conserved catabolic process that maintains cellular homeostasis by sequestering damaged organelles or misfolded proteins in the autophagosome and fusing with lysosomes for degradation and recycling (Xie and Klionsky, 2007; Klionsky et al., 2011). As a cell steward, autophagy is an essential part of host defense against pathogens (Wong and Sanyal, 2019). So far, approximately 40 autophagy related genes (Atgs) that strictly regulate this membrane trafficking process are known in yeast, and several mammalian homologs of yeast Atgs have been identified (Katherine et al., 2018).

The autophagy pathway involves two ubiquitin-like conjugation systems: Atg5-Atg12-Atg16L1 and LC3 (Atg8)-phosphatidylethanolamine (PE). The conjugation of LC3-I to PE (lipidation of LC3, LC3-II) is required for autophagosome biogenesis and is used as a standard marker of autophagy due to its location on autophagosome membrane (Mizushima et al., 2011). The Atg5-Atg12 conjugate has E3-like activity for LC3 lipidation (Hanada et al., 2007; Mizushima et al., 2011). Autophagy acts as an antiviral defense and inhibits viruses replication when challenged with some virus, such as vesicular stomatitis virus and human parainfluenza virus type 3 (Shelly et al., 2009; Ding et al., 2014; Lin et al., 2019). However, some viruses utilize the autophagy related membrane structures as a factory for replication or a shelter for escaping the host immune response, such as hepatitis B virus and influenza A virus (Zhou et al., 2009; Sir et al., 2010). Additionally, viruses can disrupt autophagy initiation to prevent viral clearance, as is the case for herpes simplex virus type 1 (HSV-1) (Orvedahl et al., 2007).

In recent years, the relationship between some aquatic viruses and autophagy has gradually been revealed, including viral hemorrhagic septicemia virus, spring viremia of carp virus, snakehead fish vesiculovirus, grouper iridovirus, largemouth bass virus, infectious kidney and spleen necrosis virus, and white spot syndrome virus (WSSV) (Garcia-Valtanen et al., 2014; Liu et al., 2015; Chen et al., 2016; Qi et al., 2016; Wang et al., 2016; Li et al., 2017). Based on current studies, the relationship between viruses and autophagy varies according to the type of virus and the host cell line. Most studies to date have focused on describing the phenomenon, information about viral induction of the autophagy signaling pathway and the autophagy–virus interaction is relatively lacking.

In this study, we focused mainly on the interaction between SGIV and autophagy in its target cells (grouper spleen, GS), and we explored the underlying interactional mechanisms.

## Materials and Methods

### Virus Strain, Cell Line, and Reagents

The GS cell line used in this study was established in our laboratory (Huang et al., 2009). GS cells were cultured in Leibovitz’s L-15 medium containing 10% fetal bovine serum (FBS, Gibco) at 28°C. The virus stock of SGIV (strain A3/12/98 PPD) was propagated in GS cells and maintained at −80°C (Qin et al., 2001). Rapamycin (Rap, R0395), Wortmannin (WM, S2758) was purchased from Selleckchem.

### Virus Infection

Unless otherwise stated, GS cells grown on 24-well culture plates (10^5^ cells/well) were infected with SGIV at multiplicity of infection of 2. For the regulating autophagy experiments, cells were pre-treated with 1 μM Rap or 1 μM WM for 2 h and then infected with SGIV according to previous studies (Li et al., 2020). For the transfected cells, SGIV infected at 24 h after transfection. At indicated hours post infection (h p.i.), RNA or protein samples were extracted as described below for further analysis.

### Western Blot Analysis

Cells were washed with phosphate buffered saline (PBS) and resuspended in immunoprecipitation (IP) lysis buffer (Invitrogen). Whole cell lysates were separated by SDS-PAGE and transferred onto a PVDF membrane (Millipore). After blocking for 1 h at room temperature in 5% skim milk or 3% bovine serum albumin (BSA) dissolved in PBS, the membrane was incubated with a primary antibody for 2 h at room temperature. The primary antibodies used in the study included anti-LC3 (Abcam, ab 58610), Beclin1 (Proteintech, 11306-1-AP), mTOR (CST, 2983T), p-mTOR (Abcam, ab109268), β-tubulin (Abcam, ab6046), p53 (SAB, 48599), and LaminB1 (Proteintech, 12987-1-AP). After washing with PBS plus 0.1% Tween 20 (PBST), the membrane was incubated with a corresponding horseradish peroxidase-coupled secondary antibody (KPL). After washing with PBST three times, immunoreactive proteins were visualized by chemiluminescence using Thermo Scientific Pierce Western Blot ECL Plus (Thermo).

### Flow Cytometry Analysis

Cellular autophagy was detected using flow cytometry according to the manufacturer’s recommendations for Cyto-ID (1:1000, Enzo). Briefly, cells were harvested and resuspended in 250 μL of Dulbecco’s PBS containing 5% FBS. Then, cells were resuspended in 250 μL of the diluted Cyto-ID Green stain solution and incubated for 30 min at 28°C in the dark. After collection by centrifugation, cells were washed with 1× assay buffer and resuspended in 250 μL of fresh 1× assay buffer. Finally, cells were analyzed using the green (FITC) channel of the flow cytometer (Beckman).

### Plasmids and Transfection Assays

The recombinant pEGFP-C1-LC3, pcDNA3.1-3 × HA-LC3, pcDNA3.1-3 × HA-Atg5, and pEGFP-MAVS were available in our laboratory (Huang et al., 2018; Li et al., 2019a, 2020). The genes of SGIV-VP48, SGIV-VP122, and SGIV-VP132 were subcloned into the vector pEGFP-C1 and pcDNA3.1-3 × HA separately. p53 from the orange spotted grouper (*Epinephelus coioides*) was subcloned into the pcDNA3.1-RFP. Lysine at position 289 of wild-type p53 was converted to asparagine by site-directed mutagenesis to create the mutant form lacking the nuclear localization signal (NLS^–^). Table 1 lists the primers used in this analysis. The constructed plasmids were subsequently verified by DNA sequencing.

**TABLE 1:** Primers used in this study.

Name	Sequence (5′–3′)
P53-F	ATGGAAGAGCAAGAGTT
P53-R	TTAGTCGCTGTCGCTCC
RFP-P53-F	GGAATTCATGGAAGAGCAAGAGTT
RFP-P53-R	GGGTACCGTCGCTGTCGCTCC
P53-NLS^–^-F	ACACCAAAAACCGAAAGAGTGCCCCGGCTGCGGCTC
P53-NLS^–^-R	CACTCTTTCGGTTTTTGGTGTGTTTGGTGCCGTTCT
VP48-F	ATGTACACTTCAAACTG
VP48-R	CTACTCAAGTTCCATCAA
GFP-VP48-F	GAAGATCTATGTACACTTCAAACTG
GFP-VP48-R	GGGGTACCCTACTCAAGTTCCATCAA
HA-VP48-F	GGGGTACCATGTACACTTCAAACTG
HA-VP48-R	CGGAATTCTCTACTCAAGTTCCATCAA
VP122-F	ATGGCACCGGGAAAAAG
VP122-R	TTATTCCAACCCCCATT
GFP-VP122-F	GAAGATCTATGGCACCGGGAAAAAG
GFP-VP122-R	GGGGTACCTTATTCCAACCCCCATT
HA-VP122-F	GGGGTACCATGGCACCGGGAAAAAG
HA-VP122-R	CGGAATTCTTTATTCCAACCCCCATT
VP132-F	ATGCATAGCGTAAAATCG
VP132-R	TTACTTTTCAAAGTACCGAG
GFP-VP132-F	GAAGATCTATGCATAGCGTAAAATCG
GFP-VP132-R	GGGGTACCTTACTTTTCAAAGTACCGAG
HA-VP132-F	GGGGTACCATGCATAGCGTAAAATCG
HA-VP132-R	CGGAATTCTTTACTTTTCAAAGTACCGAG
β-actin-RT-F	TACGAGCTGCCTGACGGACA
β-actin-RT-R	GGCTGTGATCTCCTTCTGCA
MCP-RT-F	GCACGCTTCTCTCACCTTCA
MCP-RT-R	AACGGCAACGGGAGCACTA
ICP18-RT-F	ATCGGATCTACGTGGTTGG
ICP18-RT-R	CCGTCGTCGGTGTCTATTC
VP19-RT-F	TCCAAGGGAGAAACTGTAAG
VP19-RT-R	GGGGTAAGCGTGAAGACT
LITAF-RT-F	GATGCTGCCGTGTGAACTG
LITAF-RT-R	GCACATCCTTGGTGGTGTTG

Cell transfection was carried out using Lipofectamine 2000 reagent (Invitrogen) as described previously (Li et al., 2019b). For one well of 24-well plate, cells were transfected with the mixture of 800 ng of plasmids and 2 μL of Lipofectamine 2000 diluted in serum-free Opti-MEM (Gibco). After incubation for 6 h, the medium was replaced with fresh normal medium and cells were cultured for further study. To silence endogenous LC3, GS cells were transfected with the specific siRNA (siLC3) or the same volume of the corresponding GC content negative control (NC) as described previously (Li et al., 2020).

### Subcellular Localization

To determine the subcellular localization of LC3 and p53, GS cells were seeded into glass-bottom cell culture dishes, and the constructed plasmids were transfected into GS cells as described above. At 24 h post-transfection, cells were fixed with 4% paraformaldehyde and stained with 4,6-diamidino-2-phenylindole (DAPI). LC3 and p53 were observed under a fluorescence microscope (Zeiss).

### Nuclear/Cytosol Fractionation Assay

Singapore grouper iridovirus infected cells or non-infected cells were collected and subjected to nuclear and cytosol fractionation using the Nuclear/Cytosol Fractionation Kit (BioVision) following the protocols recommended by the manufacturer. All operations are performed on ice. The separated cytoplasmic protein and nuclear protein were subjected to Western blot analysis.

### Co-immunoprecipitation Assays

To verify the interactions, the plasmids of pcDNA3.1-3 × HA-Atg5 or pcDNA3.1-3 × HA-LC3 was co-transfected with pEGFP-C1, pEGFP-MAVS, pEGFP-MAVS-CARD, pEGFP-VP48, pEGFP-VP122, or pEGFP-VP132, respectively. At 36 h after co-transfection, cells were lysed by IP lysis buffer supplemented with a protease inhibitor cocktail, then cell lysates were centrifuged at 12,000 × *g* for 5 min and the supernatant was collected for subsequent Western blot analysis and IP according to the Dynabeads^TM^ Protein G Immunoprecipitation Kit (Invitrogen). Briefly, magnetic beads were prepared and bound with anti-GFP (Abcam, ab290) for 10 min at room temperature, followed by incubation with sample containing the antigen for 30 min. After washing with washing buffer, target antigens were eluted and subjected to Western blot analysis. The primary antibodies specific for GFP and HA (Sigma, H3663) were used to detected the protein expression and interactions.

### Mass Spectrometry

Grouper spleen cells were transfected with pEGFP-C1, pEGFP-LC3, pEGFP-Atg5, respectively, in accordance with the above method and then infected with SGIV. The whole cell lysates (WCL) were precipitated with GFP antibody. IP products were detected by reversed phase liquid chromatography-mass spectrum (RPLC-MS), then the raw data was imported into Protein Discoverer 2.1 SP1 (SEQUEST HT) for analysis. The database were human proteins from Uniprot and SGIV genome annotation data set (Song et al., 2004).

### RNA Isolation and Real Time Quantitative PCR (qPCR) Analysis

For gene expression analysis, the total RNAs of cells were extracted using the SV Total RNA Isolation Kit (Promega) and reversed to synthesize the first-strand cDNA using the ReverTra Ace kit (Toyobo). Real time PCR analyses were performed using SYBR^®^ Green reagent (Toyobo) according to manufacturer’s recommendations in a Quant Studio 5 Real Time Detection System (Applied Biosystems). Primer pairs are listed in Table 1. The expression levels of target viral genes (*MCP*, *ICP18*, *VP19*, *LITAF*) were normalized to *β-actin* and calculated using the 2^–ΔΔCT^ method. All reactions were performed in triplicate, and the data are presented as relative mRNA expressed as the mean ± standard deviation (*n* = 3). One-way analysis of variance was used to evaluate the variability among treatment groups. Differences were considered statistically significant at *P* < 0.05.

### Immunofluorescence Assays

GS cells were seeded in glass-bottom cell culture dishes, then cells were treated with autophagy regulators or transfected with recombinant plasmid or siRNA. At indicated time points, cells were infected with SGIV. At 12 h p.i., cells were fixed in 4% paraformaldehyde for 1 h and permeabilized with 0.2% Triton X-100 for 15 min. After washing three times with PBS, cells were blocked with 2% BSA for 45 min and then incubated with anti-MCP serum (prepared in our laboratory) for 2 h at room temperature. Cells were washed with PBS, followed by incubation with the secondary antibody (fluorescence isothiocyanate-conjugated goat anti-rabbit immunoglobulin G, Pierce) for 1 h at room temperature. Cells then were stained with DAPI and observed under an inverted fluorescence microscope (Zeiss).

## Results

### SGIV Inhibited Autophagy Initiation in GS Cells

**FIGURE 1:**
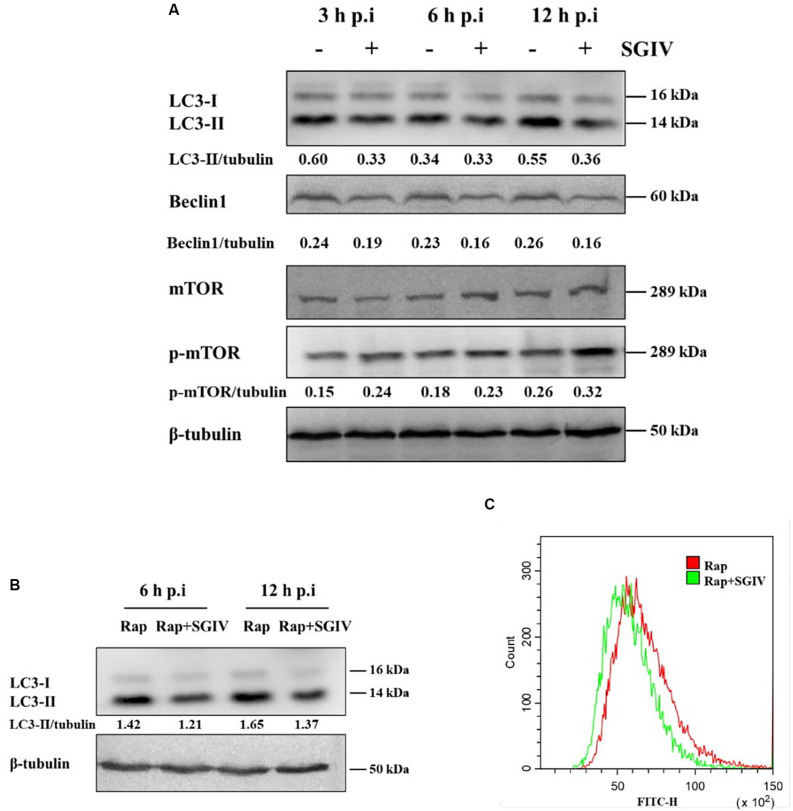
SGIV infection inhibited autophagy in GS cells. **(A)** SGIV infection altered protein levels of LC3, Beclin1, and p-mTOR as determined by Western blot analysis. β-tubulin was used as the internal control. Band intensity was calculated using Quantity-one software, and the ratio of target protein/β-tubulin was shown below the blot. **(B)** The expression of LC3 was detected by Western blot analysis in infected or non-infected SGIV cells after Rap pre-treatment. Band intensity was calculated using Quantity-one software, and the ratio of LC3-II/β-tubulin was shown below the blot. **(C)** Flow cytometry-based profiling of autophagy in infected (12 h p.i.) or non-infected SGIV cells after Rap pre-treatment. Cyto-ID dye was used to stain cells, and 1 × 10^4^ cells were collected for further positive analysis. The data were presented as the means from three independent experiment.

LC3-PE conjugates (LC3-II) are essential for membrane elongation and autophagosome formation (Mizushima et al., 2011). The electrophoresis migration rate of LC3-II in SDS-PAGE is faster than that of LC3-I. Beclin1(Atg6) plays a central role in initiation of the autophagy pathway by marking membranes to form the first double membrane structure, the phagophore (Kang et al., 2011). In this study, LC3 and Beclin1 protein levels were firstly detected by Western blot at different time points after SGIV infection. As shown as in Figure 1A, LC3-II and Beclin1 expression were both decreased in SGIV infected cells compared with the non-infected cells, especially at 3 h p. i. and 12 h p. i.

The mammalian target of Rapamycin (mTOR) is generally considered to be an inhibitor of autophagy, and the levels of mTOR and phosphorylation of mTOR were detected in this study. As shown as in Figure 1A, the level of phosphorylated mTOR (p-mTOR, S2448) were increased at 3 h, 6 h, and 12 h p. i., which suggested that SGIV infection might decrease autophagy through unlocking mTOR activity to some extent. To further ascertain the inhibition of SGIV replication on autophagy, we verified the result by means of the positive inducer Rap. In cells which pre-treated by Rap and infected with SGIV, LC3-II level decreased at 6 h p.i. and 12 h p.i. compared with the cells only pre-treated by Rap (Figure 1B), indicating that SGIV infection impeded autophagy activity to a certain extent. The measurement of autophagy with Cyto-ID dye also indicated that SGIV infection reduced the number of autophagy positive cells (Figure 1C).

### SGIV Infection Increased Cytoplasmic p53 to Inhibit Autophagy

**FIGURE 2:**
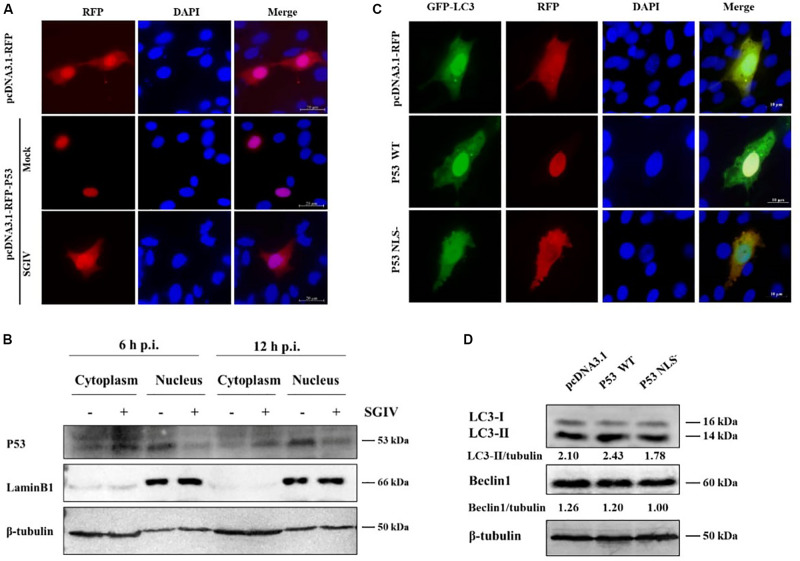
SGIV increased cytoplasmic p53 level to inhibit autophagy. **(A)** SGIV infection altered the subcellular localization of p53. The transfected cells were fixed at 3 h p. i. **(B)** SGIV infection increased cytoplasmic p53 and decreased nuclear p53 levels. β-tubulin and LaminB1 were the internal references for cytoplasmic and nuclear extracts, respectively. **(C)** The cytoplasmic p53 (NLS^–^) reduced the clustering of GFP-LC3. pcDNA3.1-RFP, RFP-p53 WT, and RFP-p53 NLS^–^ were co-transfected with GFP-LC3 plasmid into GS cells for 24 h, and stained with DAPI. **(D)** Subcellular localization of p53 affected the level of autophagy-related proteins. Band intensity was calculated and ratios of target protein/β-tubulin were assessed. The data were presented as the means from three independent experiment.

Research shows p53 affects autophagy activity differently in different locations, which is characterized by cytoplasmic p53 inhibiting autophagy but nuclear p53 promoting autophagy (Tasdemir et al., 2008a). In this study, p53 was distributed mainly in the nucleus in non-infected cells, but it was transferred to the cytoplasm upon SGIV infection (Figure 2A). We verified these results by nuclear/cytosol fractionation of p53 at 6 h and 12 h after SGIV infection. In SGIV infected cells, cytoplasmic p53 level was increased and nuclear p53 level was decreased compared with levels in non-infected cells (Figure 2B). To ascertain whether p53 from grouper has a similar function in autophagy as that in mammals, a p53 mutant with a mutation (NLS^–^) was generated by converting lysine codons at positions 289 to asparagine using site-directed mutagenesis. The wild type p53 (p53 WT), p53 NLS^–^, and the empty vector were co-transfected with GFP-LC3 into GS cells. The subcellular localization analysis showed that little LC3 accumulated in the cells transfected with p53 NLS^–^, indicating that cytoplasmic p53 inhibited autophagy (Figure 2C). Meanwhile, we detected the effect of p53 WT and p53 NLS^–^ on autophagy related proteins. As shown in Figure 2D, p53 WT increased the level of LC3-II, while cytoplasmic p53 decreased LC3-II and Beclin1, compared with transfected empty vector cells. Above all, SGIV infection leads to the transfer of p53 from the nucleus to the cytoplasm, which might be one of strategies to inhibit autophagy.

### SGIV-VP48, VP122, and VP132 Interacted With Atg5 to Inhibit Autophagy Activity

**FIGURE 3:**
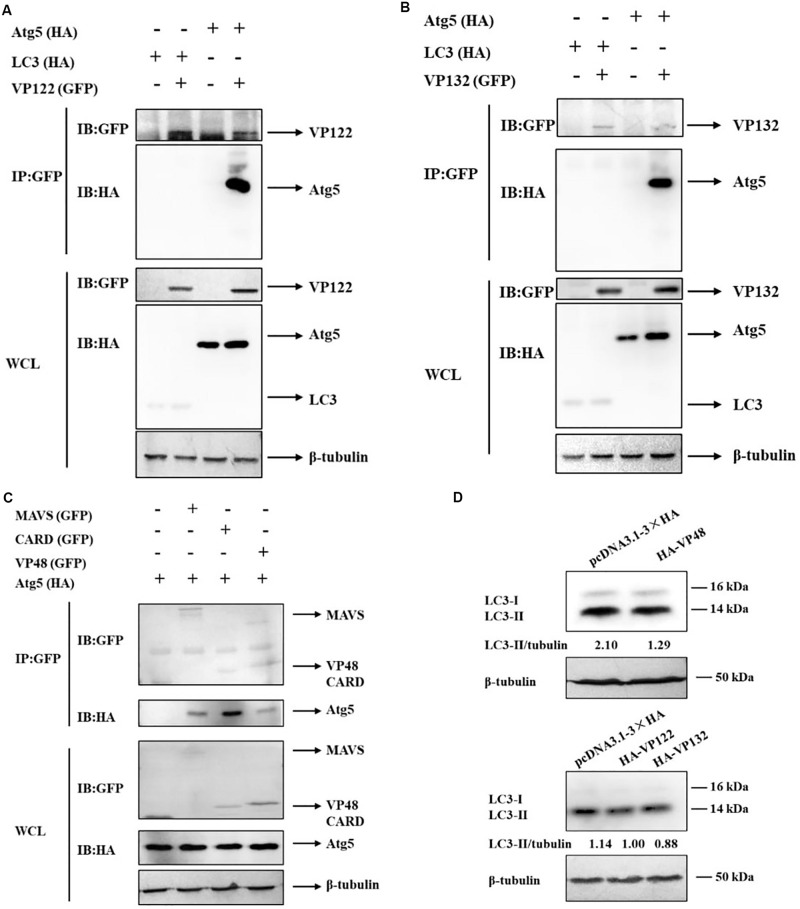
SGIV-VP48, VP122, and VP132 interacted with Atg5 and their ectopic expression decreased the level of LC3-II. **(A)** HA-Atg5 interacted with GFP-VP122. Whole cell lysates (WCL) transfected with GFP-VP122 and HA-Atg5 or HA-LC3 were subjected to immunoprecipitation (IP) and immunoblotting (IB) with indicated antibodies. **(B)** HA-Atg5 interacted with GFP-VP132. WCLs of cells transfected with GFP-VP132 and HA-Atg5 or HA-LC3 were used for IP and IB with indicated antibodies. **(C)** HA-Atg5 interacted with GFP-MAVS, GFP-CARD, and GFP-VP48. WCLs of cells transfected with HA-Atg5 and GFP-MAVS, GFP-CARD, or GFP-VP48 were used for IP and IB with indicated antibodies. **(D)** VP48, VP122, and VP132 decreased the level of LC3-II. Cells transfected with HA-VP48, HA-VP122, or HA-VP132 were collected for Western blot analysis. β-tubulin was used as the internal reference. Band intensity was calculated using Quantity-one software, and ratios of LC3-II/β-tubulin were assessed. The data were presented as the means from three independent experiment.

It has been reported that some viral proteins can bind to autophagy-related proteins, such as Atg5 and LC3, to hijack the autophagy process (Guevin et al., 2010). In this study, viral proteins as potential Atg5 or LC3 interactants were analyzed by co-IP and mass spectrometry. ORF122 (VP122) and ORF132 (VP132) of SGIV were identified in the IP products of GFP-Atg5 and GFP-LC3 (Table 2). It also has been reported that Atg5 can bind to the CARD domain of mitochondrial antiviral signaling protein (MAVS) and down-regulate innate antiviral immunity (Jounai et al., 2007). Based on the proteins known to be encoded by SGIV, we found that VP48 encodes a CARD domain protein (Song et al., 2004). Herein, we verified the interaction of Atg5 with VP122, VP132, MAVS, the MAVS-CARD domain, and VP48. We also verified the interaction of LC3 with VP122, VP132. By detecting the IP products of GFP-VP122 and GFP-VP132, we found that HA-Atg5 readily interacted with GFP-VP122, GFP-VP132 upon transient overexpression in GS cells (Figures 3A,B). The results showed that Atg5 interacted with MAVS and VP48, and more specifically with the CARD domain (Figure 3C). However, LC3 did not directly interact with VP122 and VP132.

**TABLE 2:** Summary of the proteomic profile of peptides detected following LC3, Atg5 immunoprecipitations in GS cells.

Protein accessions	Protein descriptions	*Q*-value	GFP-C1	GFP-LC3	GFP-Atg5
A0A484BZB5	Autophagy-related protein 3	6.21E-06	Filtered	14972.775	Filtered
Q5YFD3	Uncharacterized protein (ORF132R)	8.16E-06	Filtered	1	26748.252
Q5YFE3	Uncharacterized protein (ORF122L)	5.42E-05	Filtered	4418.675	17621.111

*The protein accessions of Candidate molecules in Uniprot database were listed and the detailed information of these proteins can be queried in Uniprot website (https://www.uniprot.org/).*

Atg5 plays an important role in the conversion of LC3-I to LC3-II, we speculated that VP48, VP122, and VP132 might competitively bind with Atg5, thereby blocking of the conversion of LC3-I to LC3-II. In this study, we evaluated the effect of VP48, VP122, and VP132 on the conversion of LC3-I to LC3-II and found that the level of LC3-II was decreased in VP48, VP122, and VP132 overexpressed cells (Figure 3D). To sum up, viral proteins can bind to Atg5 and downregulate levels of the autophagosome-associated form of LC3, which might be another strategy that allows SGIV to inhibit autophagy.

### Inducing Autophagy by Rap Decreased SGIV Replication, Whereas Inhibiting Autophagy by WM Promoted SGIV Replication

**FIGURE 4:**
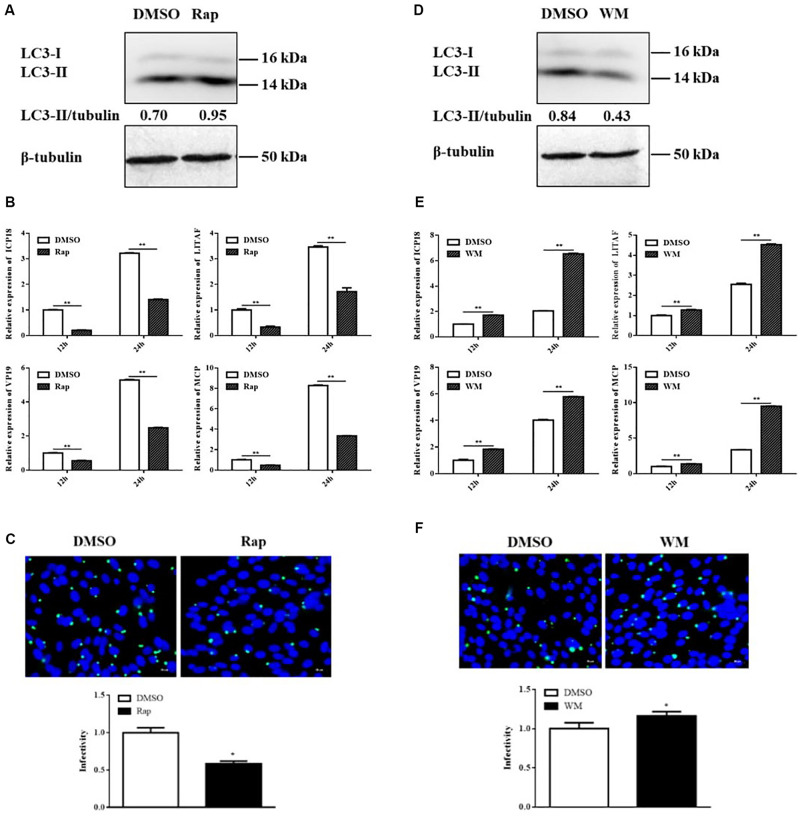
Rap decreased SGIV replication, whereas WM promoted SGIV replication. GS cells were pretreated with Rap or WM or the same dosage of DMSO for 2 h prior to SGIV infection, then the infected cells were collected and analyzed by qPCR and immunofluorescence assay (IFA). **(A,D)** The LC3 expression in Rap or WM pretreated cells. **(B,E)** The relative expression of MCP, ICP18, VP19, and LITAF in SGIV infected cells. The β-actin gene was used as the internal reference for qPCR. **(C,F)** The infected cells were analyzed at 12 h p.i. by IFA using anti-MCP antibody (green). Nuclei were stained by DAPI (blue). The viral infectivity of control (DMSO treated) cells was set as 1. The data were presented as the means from three independent experiment, *p < 0.05, **p < 0.01.

Considering that SGIV inhibited autophagy, we deduced that autophagy might play an antiviral role upon SGIV replication. To verify this supposition, we treated the cells with Rap or WM for 2 h to induce or inhibit autophagy, respectively. The results showed that the LC3-II expression was increased in Rap-treated and decreased in WM-treated cells accordingly (Figures 4A,D). The pre-treated cells then were inoculated with SGIV to detect the effect of autophagy on viral replication. In this study, qPCR detection of the expression of viral genes, including *MCP*, *ICP18*, *VP19*, and *LITAF*, showed that they were all significantly decreased in Rap pre-treated cells compared to DMSO treated cells (Figure 4B). In addition, the immunofluorescence assay for SGIV major capsid protein (MCP) showed that MCP protein synthesis decreased after Rap treatment (Figure 4C). Conversely, the expressions of viral genes and MCP protein synthesis increased in WM pre-treated cells (Figures 4E,F). These results indicated that autophagy acts as a defense mechanism upon SGIV replication.

### Overexpressing LC3 Decreased SGIV Replication, Whereas Silencing LC3 Promoted SGIV Replication

**FIGURE 5:**
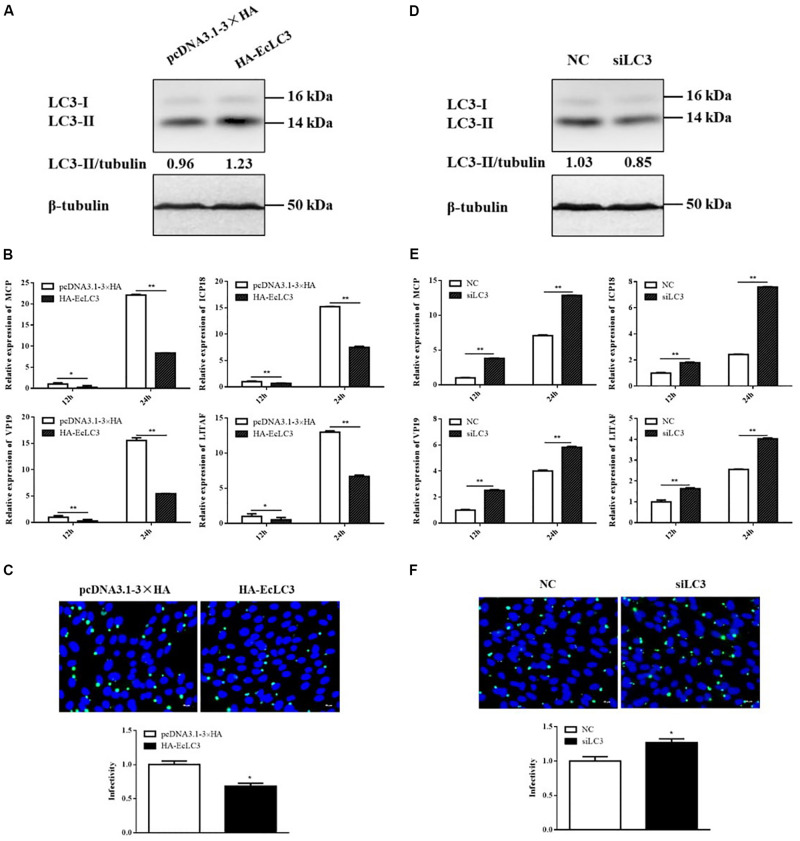
LC3 overexpression decreased SGIV replication, whereas LC3 knockdown promoted SGIV replication. GS cells were transfected with HA-LC3 or the vector, siLC3 or the negative control (NC), and then infected with SGIV. Viral replication was analyzed by qPCR and IFA. **(A,D)** The LC3 expression in transfected cells. **(B,E)** The relative expression of MCP, ICP18, VP19, and LITAF in SGIV infected cells. The β-actin gene was used as the internal reference for qPCR. **(C,F)** The infected cells were analyzed at 12 h p.i. by IFA using anti-MCP antibody (green). Nuclei were stained by DAPI (blue). The viral infectivity of control cells was set as 1. The data were presented as the means from three independent experiment, *p < 0.05, **p < 0.01.

In addition to chemical regulators, we also explored the effect of autophagy on SGIV replication by overexpressing and silencing LC3. The overexpression and interference effects were reflected by LC3 expression. As shown as in Figures 5A,D, overexpressing or silencing LC3 increased or decreased LC3-II level accordingly. The expressions of viral genes, including *MCP*, *ICP18*, *VP19*, and *LITAF*, were all significantly decreased in cells overexpressing LC3 (Figure 5B). The immunofluorescence of MCP showed that MCP protein synthesis also decreased in cells overexpressing LC3 (Figure 5C). In contrast, both viral gene expression and protein synthesis increased after silencing LC3 with siRNA (Figures 5E,F). These results indicated that LC3 exerted the antiviral role of autophagy against SGIV replication.

## Discussion

Autophagy is an essential process required to maintain cellular homeostasis. This process can be induced by various cellular stresses, including nutrient deprivation, oxidative stress, the unfolded protein response, and pathogen invasion (Deretic et al., 2013). A series of studies has demonstrated that some viral infections can alter the autophagy level, which functions as either a pro-viral or antiviral pathway, depending on the virus and its host cells (Lennemann and Coyne, 2015). In this study, we found that SGIV infection inhibited autophagy in GS cells. The strategies by which SGIV inhibited autophagy were demonstrated, which included causing the transfer of p53 from the nucleus to the cytoplasm and encoding some viral proteins that interact with Atg5 to block LC3 lipidation. As a defense mechanism, cellular autophagy and the key protein LC3 play the antiviral role in SGIV replication.

LC3-II is the protein marker that is reliably associated with completed autophagosomes (Klionsky et al., 2008). Beclin1, which forms a complex with Vps34, the class III phosphatidylinositol 3-kinase, is an important protein for autophagy initiation (Shrivastava et al., 2012). In this study, SGIV infection significantly decreased the LC3-II and Beclin1 protein levels. The mammalian target of Rap (mTOR) is generally considered to be an inhibitor of autophagy induction by inhibiting the phosphorylation of ULK1(Atg1) (Sarkar et al., 2009; Wong et al., 2013). Contrary to the trend of LC3-II and Beclin1, the p-mTOR level increased with the extension of infection time. Thus, we preliminarily speculated that SGIV inhibited autophagy and that the pathway was mTOR-dependent. Additional evidence for this inhibition was obtained using an autophagy inducer. In the case of Rap treatment, SGIV also decreased the LC3-II level and the number of autophagy positive cells. These results indicated that SGIV infection inhibited autophagy in GS cells. Similar phenomena have been reported for pseudorabies virus (Sun et al., 2017).

The cytoplasmic and nuclear p53 have different effects on autophagy (Tasdemir et al., 2008a; Morselli et al., 2011). The present study showed that SGIV replication led to the transfer of p53 from the nucleus to the cytoplasm. Moreover, the accumulation of LC3 (LC3-II) was inhibited in the p53 NLS^–^ cells, indicating that grouper p53 has functions similar to those of mammals in regulating autophagy. However, there are various mechanisms by which p53 can suppress autophagy directly or indirectly, independent or dependent on mTOR (Tasdemir et al., 2008b; Morselli et al., 2011; Tang et al., 2015). The specific mechanism by which p53 affects autophagy in SGIV infection needs further study. Interestingly, some viruses manipulate autophagy through interaction with autophagic proteins (Pirooz et al., 2014; Lennemann and Coyne, 2015; O’Connell and Liang, 2016). For example, human immunodeficiency virus-1 (HIV-1) precursor protein Gag interacts with LC3, which augments Gag processing and HIV yields (Kyei et al., 2009). Moreover, HIV-1 accessory protein Nef and HSV-1 ICP34.5 block autophagosome maturation through interaction with Beclin1 (Orvedahl et al., 2007; O’Connell and Liang, 2016). In our study, we demonstrated that SGIV VP48, VP122, and VP132 interacted with Atg5, which impeded the conversion of LC3-I to LC3-II. In addition, our previous studies have shown that Atg5 is a pro-viral factor during SGIV infection (Li et al., 2019a). Therefore, Atg5 is an important target for SGIV to hijack autophagy. Similar results have been reported for hepatitis C virus (Guevin et al., 2010). Whether there are other targets that SGIV utilizes to inhibit autophagy remains to be determined. Recent studies have shown that SGIV is equipped with some viral proteins to regulate apoptosis and escape the host immune and inflammation response (Huang et al., 2008; Yu et al., 2017). Our study showed that some viral proteins are involved in the autophagy pathway, which might be also related to the pathogenesis mechanisms of SGIV.

Previous studies have shown that the effect of autophagy on virus replication is virus and cell-type specific (Mohl et al., 2012; Deretic et al., 2013; Zhang et al., 2014; Sparrer and Gack, 2018). In our study, SGIV replication was inhibited by activation of autophagy by Rap or overexpression of LC3, whereas inhibition of autophagy by WM or silencing LC3 promoted viral replication. These findings indicate that SGIV cannot use autophagy-related membrane structures as viral replication sites. Similarly, GABARAP (a member of the Atg8 family) suppressed WSSV replication in hematopoietic tissue cells (Chen et al., 2016).

**FIGURE 6:**
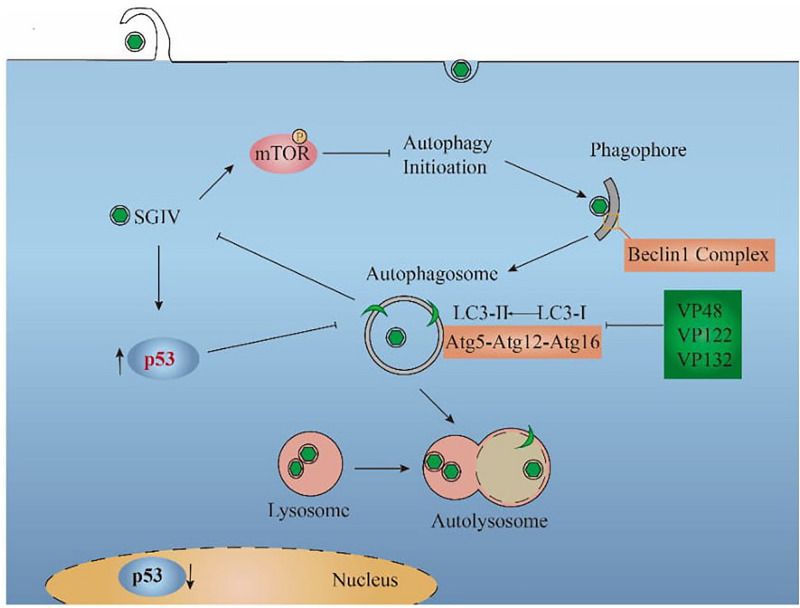
Proposed model for the interaction between SGIV and autophagy. SGIV infection inhibited autophagy initiation, which is mTOR-dependent. The inhibition mechanisms of SGIV include increasing the cytoplasmic p53 level and encoding viral proteins (VP48, VP122, VP132), which bind Atg5 to block the LC3 conversion. From the perspective of the host cell, autophagy pathway decreased SGIV replication.

In Figure 6, the relationship between SGIV and autophagy was summarized based on the results of this study. SGIV infection inhibited autophagy by increasing the cytoplasmic p53 level and encoding VP48, VP122, and VP132, which bind Atg5 to affect the LC3 conversion. As an antiviral defense, autophagy pathway and the autophagic key protein LC3 suppressed SGIV replication. Further discoveries in the area of autophagy-mediated host defenses will help to provide new antiviral strategies.

## Data Availability Statement

The datasets generated for this study are available on request to the corresponding author.

## Author Contributions

QQ and JW designed the experiments. CL performed the majority of the experiments, analyzed data, and wrote the manuscript. LW, JL, and YY contributed to experimental suggestions. YH and XH helped to design the experiments. All authors revised the manuscript.

## Conflict of Interest

The authors declare that the research was conducted in the absence of any commercial or financial relationships that could be construed as a potential conflict of interest.
